# Obsolescence effects in second language phonological networks

**DOI:** 10.3758/s13421-023-01500-9

**Published:** 2023-12-04

**Authors:** Eva Maria Luef

**Affiliations:** 1 Institute of English and American Studies, University of Hamburg, Von-Melle-Park 6, 20146 Hamburg, Germany; 2 Department of English and ELT Methodology, Faculty of Arts, Charles University, nám. Jana Palacha 2, 116 38 Prague 1, Czech Republic

**Keywords:** Network obsolescence, English as a second language, Network evolution, Phonological network, Preferential attachment

## Abstract

Phonological networks are representations of word forms and their phonological relationships with other words in a given language lexicon. A principle underlying the growth (or evolution) of those networks is preferential attachment, or the “rich-gets-richer” mechanisms, according to which words with many phonological neighbors (or links) are the main beneficiaries of future growth opportunities. Due to their limited number of words, language lexica constitute node-constrained networks where growth cannot keep increasing in a linear way; hence, preferential attachment is likely mitigated by certain factors. The present study investigated obsolescence effects (i.e., a word’s finite timespan of being active in terms of growth) in an evolving phonological network of English as a second language. It was found that phonological neighborhoods are constructed by one large initial lexical spurt, followed by sublinear growth spurts that eventually lead to very limited growth in later lexical spurts during network evolution. First-language-given neighborhood densities are rarely reached even by the most advanced language learners. An analysis of the strength of phonological relationships between phonological word forms revealed a tendency to incorporate phonetically more distant phonological neighbors at earlier acquisition stages. Overall, the findings suggest an obsolescence effect in growth that favors younger words. Implications for the second-language lexicon include leveraged learning mechanisms and learning bouts focused on a smaller range of phonological segments, and involve questions concerning lexical processing in aging networks.

## Introduction

**Fig. 1:**
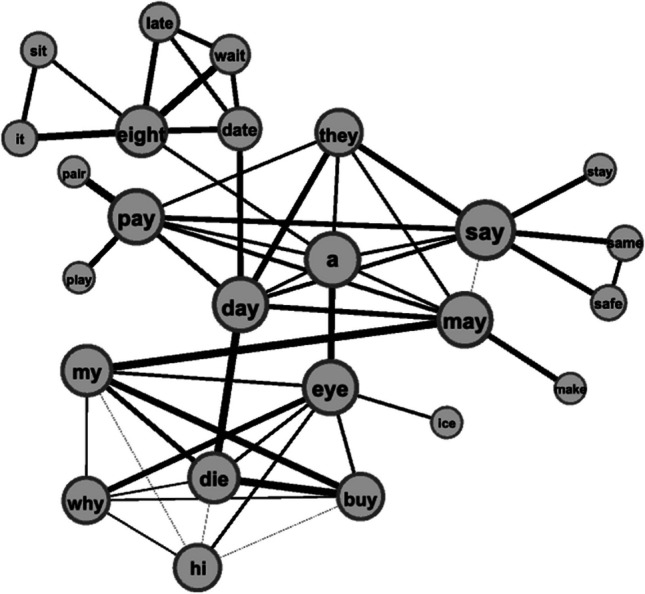
Part of a phonological network of the A1 lexicon of (British) English as a second language. Larger nodes have more phonological neighbors (i.e., denser neighborhoods). Thicker edges indicate a closer phonological relationship between two neighbors. The network was drawn with *Gephi*

Lexical memory is governed by similarity principles, with the storage and retrieval of words being influenced by the number of similar neighbors, which in their entirety constitute similarity neighborhoods (e.g., Buchwald, 2011; Mirman & Magnuson, 2008). Phonological neighborhoods represent an accumulation of words that are similar in word form to one another, and they play crucial roles in speech perception and production (Vitevitch & Luce, 2016). A tool well suited to model the dyadic relationships between words, their neighborhoods, and the phonological similarities between neighbors is network science (Barabási, 2016). In phonological networks, phonological word forms represent the nodes, and a link (i.e., edge) is placed between them in case of phonological similarity. Definitions of phonological similarity may vary (Castro & Vitevitch, 2022). For instance, phonological associations and misperceived words may be used as the basis for links between word forms (Castro & Vitevitch, 2022), in addition to the more traditional psycholinguistic metric of phonological neighbor, the one-segment difference between words (i.e., the Levenshtein distance, see Levenshtein, 1966; Vitevitch, 2008, 2021). It is an option to weight edges, i.e., to define the strength of the relationship between neighbors. The majority of phonological networks are unweighted (e.g., Chan & Vitevitch, 2010; Fourtassi et al., 2020; Luef, 2022a; Siew & Vitevitch, 2020a; Vitevitch, 2008); those that are base the calculation of weights on the number of participants who report phonological neighbors in certain association and misperception tasks (Castro & Vitevitch, 2022). The one-segment neighborhood has been a robust and reliable predictor for speech processing, both weighted (e.g., Fricke et al., 2016; Goldrick et al., 2013) and – more commonly - unweighted (e.g., Vitevitch & Luce, 2016). Thus, it makes sense to enhance and further quantify the one-segment metric by computing a theoretically grounded phonetic distance measurement between the neighbors. For instance, the English words “bat” and “pat” are phonologically closer neighbors than “bat” and “sat” in terms of the number of shared phonetic features. Figure 1 illustrates a part of a phonological network, with variable edge strengths indicating phonetic distances. Such a method can result in a pattern encompassing all word form relationships within a given lexicon. Psycholinguistic research has proven that the construct of a phonological network and a word’s location in it influences the speed and accuracy of speech recognition (Chan & Vitevitch, 2009; Siew & Vitevitch, 2016; Vitevitch et al., 2014) and speech production (Chan & Vitevitch, 2010), as well as lexical learning (Fourtassi et al., 2020; Luef, 2022a; Siew & Vitevitch, 2020a).

Network evolution (or network growth) aims to model processes that can explain changes in network structure over developmental time periods (Albert & Barabási, 2002). Various network growth models have been proposed that can account for growth dynamics in a range of social, technological, biological, and cognitive networks (Hills et al., 2009; Newman et al., 2006; Toivonen et al., 2009). Phonological networks have been classified as Barabási-Albert network growth models (Barabási & Albert, 1999), which have at their core a process called preferential attachment or the “rich-gets-richer” growth mechanism (Fourtassi et al., 2020; Luef, 2022a; Siew & Vitevitch, 2020a). Preferential attachment dictates that growth is more likely to take place in nodes with many neighbors (i.e., high-degree nodes) than in those with few neighbors (i.e., low-degree nodes). According to this mechanism, words with many phonological neighbors (i.e., in dense neighborhoods) have an advantage in acquiring new neighbors during network evolution. For instance, the English word “cat” with 50 neighboring words (or *degree = 50* in network terms) has a higher probability of gaining a new neighbor in a new round of lexical learning than the word “ghost,” which has only 14 neighbors in English (see Marian et al., 2012, for neighborhood density statistics). Over time, preferential attachment leads to scale-free structuring on the macro level of phonological networks, with few “hubs” – or nodes with a very large number of neighbors – co-existing with a large majority of low- or no-degree nodes (Barabási & Albert, 1999).

Preferential attachment in network evolution is predisposed upon the requirement that a sufficient number of nodes is available so that nodes can add an ever-growing group of neighbors at any future round of node additions. In phonological networks growth is naturally limited by the available number of words that are contained in the lexicon of a language. Linear preferential attachment is not possible throughout all developmental stages, and growth patterns are expected to turn sublinear when the pool of new nodes becomes depleted. Eventually, growth will slow to a near or complete halt, if there is no form of node augmentation (Rossmannek & Rank, 2020; Wu et al., 2013). Node-constrained preferential attachment networks are predicted to show larger initial growth spurts, with consecutive spurts becoming smaller in size. The first spurt offers the greatest possibilities for a node to add neighbors, and each following growth spurt faces increasingly limited options for expansion. What results from this dynamic is a mechanism that attributes the largest growth opportunities to newly introduced nodes as they enter a lexical system. At each growth spurt that the network undergoes, the newest nodes are endowed with the greatest growth opportunities due to the fact that growth options run out for older nodes. Such an effect is known as “obsolescence” or “aging” in networks (Safdari et al., 2016), and it is most commonly defined as a node’s “finite time span of being active” (Zhu et al., 2003, p. 1). This means that a node’s ability to grow neighbors is limited by its age (Dorogovtsev & Mendes, 2000; Zhu et al., 2003). Nodes may not retain their full capacity for attracting new connections throughout all stages of network evolution, but tend to lose their growth relevance over time, frequently attributing more growth opportunities to younger nodes (Hu et al., 2021). In these aging networks, preferential attachment becomes restricted to the subset of recently added nodes, with older nodes not being able to join up with incoming new nodes any longer. Such a growth dynamic can lead to the opposing mechanism of preferential attachment by virtue of node exhaustion (Safdari et al., 2016). This exact mechanism has been identified in evolving phonological networks where it has been termed “inverse preferential attachment” (Siew & Vitevitch, 2020a, b).

Growth in phonological networks displays a special feature: a switch from the initial preferential attachment to the inverse variant of the mechanism at more advanced lexical acquisition stages (Luef, 2022a; Siew & Vitevitch, 2020a, b). Siew & Vitevitch (2020a) first demonstrated this change in network growth strategies in their study of first-language English- and Dutch-speaking children’s lexical acquisition. They found that word forms with many phonological neighbors tended to attract the majority of new neighbors at initial stages of language acquisition (termed the “PATT” mechanism), but that word forms with few phonological neighbors acquired the majority of incoming words at later acquisition stages (termed the “iPATT” mechanism). The “PATT-iPATT switch” refers to the point in language acquisition when growth probability is higher for low-degree than for high-degree nodes in the phonological network. The switch has been suggested to result from the fact that the lexicon has exhausted all neighbor possibilities and there are no words left to be added to the dense neighborhoods; consequently, growth is directed to the sparser regions of the lexicon. The switch may be a vehicle to facilitate lexical processing by avoiding an over-densification of neighborhoods, which poses difficulties to speech recognition (Luef, 2022a; Siew & Vitevitch, 2020a). Obsolescence effects in evolving phonological networks could provide a relatively simple explanation for the presence of inverse preferential attachment: each word maximizes growth at its entry point in the lexicon before it runs out of options to expand its neighborhood. Paired with the lexical growth dynamic of high-degree words being acquired first (Luef, 2022b), this leaves later growth spurts with only low-degree nodes to acquire.

Not all neighbors are equal in phonological terms and phonetic distance could have an influence on phonological neighborhood growth, similar to neighborhood effects in speech processing (e.g., Fricke et al., 2016; Goldinger et al., 1989). Each word in a lexicon creates a phonological representation in lexical memory, and words that share segments share parts of their representations (Stamer & Vitevitch, 2012; Storkel et al., 2006; Vitevitch & Sommers, 2003). With each new word, a new representation must be formed and this is easier if a similar representation already exists that can partly be shared with the new word, hence the facilitation of word learning in the case of phonological similarity (Jusczyk et al., 1994; Storkel et al., 2006). This effect could be accelerated by phonetic overlap between word forms. In weighted evolving networks where various link strengths appear, preferential attachment may cause closer neighbors to be preferentially acquired at the expense of more distant neighbors (e.g., Rui & Ban, 2012; Ruiz et al., 2020; Topirceanu et al., 2018). Node obsolescence in phonological networks may be mediated by edge-wise preferential attachment so that greater phonetic similarity is more advantageous for word-form learning and could lead to the effect that closer neighbors are preferentially acquired during phonological network evolution.

This study will focus on obsolescence effects of degree and weighted degree centralities in an evolving second-language (L2) phonological network of English. In addition, the acquisition efficiency of the first language (L1)-given neighborhood densities by L2 learners will be investigated to see whether L2 learners exhaust their vocabulary options in dense neighborhoods before they move on to acquire lower-density words. The following research questions will be investigated:

1. Do growth spurt sizes differ over a node’s life span in a network? Which growth spurt adds the largest proportion of new neighbors to a phonological neighborhood?
2. Does the exhaustion of possible phonological neighbors in a language play a role?
3. Does preferential attachment favor the acquisition of phonetically closer neighbors in initial growth spurts?

Understanding the specific dynamics behind preferential attachment-driven growth of lexical networks can be key to predicting the cognitive resources needed for learning words and organizing lexical memory at the different acquisition phases of second-language learning.

## Methods

First, the vocabularies of different proficiency or age-of-acquisition (AoA) stages were established for English as a second language (ESL). Growth spurts occurring in between those stages were then analyzed to determine (a) the number of phonological neighbors added to a word (“growth spurt size analysis”), (b) how dense a word’s neighborhood has become in relation to L1-given neighborhood density (“saturation analysis”), and (c) the chronological addition of close versus distant phonological neighbors to a word’s neighborhood (“phonological distance analysis”).

Second-language research involving European languages generally follows the guidelines published by the “Common European Framework of Reference for Languages” (CEFR, Council of Europe, 2018), which divides the language learning phase into six distinct proficiency stages, starting at A1, A2, B1, B2, and leading up to the most advanced stages of C1 and C2. In addition, there is a recognized A0 level, which marks beginning learners without any knowledge of a particular language. The proficiency levels correspond to AoA stages in language development research and will be referred to as “AoA” and stages 0–6 in the present study (see Table 1). Stage A0 or AoA-0 is not defined in terms of a lexicon but functions as a theoretical starting point for lexical acquisition.

**Table 1:** Common European Framework of Reference for Languages (CEFR) proficiency levels in second languages and their age of acquisition (AoA) correspondences

CEFR proficiency levels	AoA stages
A0	0
A1	1
A2	2
B1	3
B2	4
C1	5
C2	6

Lexical data for the AoA stages were collected from the website *English Profile*, which is an interdisciplinary program led by Cambridge University Press and Cambridge English Language Assessment that describes vocabulary and grammatical knowledge for all CEFR proficiency stages of second-language English. The vocabulary word lists can be accessed at the sub-section *English Vocabulary Profile* (EVP; see, e.g., Harrison, 2015), and they stem primarily from the Cambridge Learner Corpus, the largest corpus of adult second-language English, but are supplemented with data coming from other sources (see Capel, 2015, for more information). The EVP provides word lists for British and American English; for the present study American data were collected. A total of six word lists were gathered, one for each CEFR proficiency level. The word lists contained mainly lemmata. Cleaning and sorting of the data included the merging of duplicate word forms as one phonological form (e.g., noun and verb “span”), splitting of phrases and clitics into their constituent parts (e.g., “it’s” ➔ “it”, “is”), and removal of abbreviations (e.g., “DVD”). The word lists contained all parts of speech: the averages across all CEFR levels were 47% nouns, 20% verbs, 21% adjectives, 7% adverbs, 1% determiners, and 3% prepositions.

As a next step, each word list was processed with an automatic lemmatizer (R package 'udpipe'; see Wijffels, 2019) to eliminate inflections, with the exception of only a few, extremely frequent inflected words that were kept in the final dataset. These included inflections of the verbs “be” and “have” and of the adjective “good” (i.e., “better,” “best”). Two word lists were compiled per AoA stage: (1) the entire vocabulary of that stage, (2) the unique vocabulary newly acquired at that stage (see Table 3 for an overview of the final vocabulary sizes for each AoA stage).

Next, the word lists were copied into the open-access electronic resource CLEARPOND database (Marian et al., 2012) in order to obtain lexical statistics of the word forms, such as frequency rate and phonological neighborhood density of L1-American English, as well as phonological SAMPA transcriptions. These are machine-readable phonetic transcriptions (Wells, 1997), for instance “think” is SAMPA-transcribed into “TINk” and can be processed by software such as R, which cannot easily handle traditional international phonetic transcriptions (IPA), such as “θɪŋk”. The SAMPA transcriptions provided by CLEARPOND had to be edited in the following ways: the dots in between symbols were removed and double-letter/number symbols, such as “r0” or “36” were replaced with unique single-digit numbers. For accuracy, only those words contained in the CLEARPOND corpus were included in the final word lists. This meant removal of a small percentage of the overall lexical data (= 3.9% across all levels).

In case of homophonous word forms (e.g., “two”-“too” or “plain”-“plane”), average lexical frequency rates were calculated. For instance, the word “plain” has a lexical frequency rate of 21.8, its homophonous equivalent “plane” of 95.5, resulting in an average lexical frequency rate of 58.6 for the SAMPA word form /pleIn/. All frequency rates were log-transformed using the formula LOG(x+1) in Excel to account for zero values.

Phonological length of words was determined by counting the number of phonemes contained (with the Excel function *len*) in the IPA-transcribed word form. IPA transcriptions were obtained from the online database “tophonetics” set to American English.

Finally, all SAMPA transcriptions were tested against one another in terms of Levenshtein distance between them, so that all one-segment neighbors (substitution, deletion, addition) could be identified within each of the six word lists. This was done with an Oracle 12c *Big-Data-Lite* database (Bryla, 2015) with the function *edit_distance* of the package ‘utl_match.’ Once all one-segment neighbors had been identified as phonological neighbors, these words were further analyzed in terms of their phonetic distances. For this, the ‘alineR’ package in “R” was used, which provides feature-weighted phonetic similarity scores between word forms based on a number of phonetic features, including, for instance, dental, palato-alveolar, nasal, aspirated, long, and central, among others (see Downey et al., 2017). The ALINE algorithm tests the presence/absence of a number of features in two compared phonological word forms and calculates a distance score ranging between “0” (= no distance, homophones) and “1” (= greatest distance). While the ALINE algorithm was developed for comparison of two words and, ultimately, to determine relationships between languages (Kondrak, 2000), it is well suited to be used on phonological neighbors differing by only one segment (Downey et al. 2017). For the present study, all previously identified phonological neighbors in all word lists were transcribed according to the international phonetic alphabet (IPA) in order for alineR to be able to read them. Then the algorithm computed a phonetic distance between them. Since phonological neighbors in networks are more intuitively characterized by similiarity scores rather than distance scores, the alineR output was reverse rescaled so that “1” would represent perfect phonetic similarity. The similarity scores were then converted to integer numbers (multiplying them by 100) for easier processing in the network program for further analysis (see Table 2 for examples).

**Table 2:** Example of alineR results and rescaling of phonetic distances

Word “a”	Neighbor	alineR output	Rescaled
"eɪ"	"deɪ" (day)	0.36842	63
"eɪ"	"eɪt" (eight)	0.36842	63
"eɪ"	"aɪ" (eye)	0.16666	83

The six CEFR proficiency levels for second languages represent specific achievements during the course of language learning. They are more or less arbitrary timepoints that are solely characterized by language learning progress, rather than the actual age of the learners. Therefore, the AoA stages as defined by the present study correspond to “plateaus” of linguistic ability, and the focus of the present study is the investigation of how the phonological neighborhood densities change between these “plateaus”’ in response to previously occurring growth phases. A detailed look at lexical knowledge at each of these AoA stages can reveal which words were able to benefit from growth during a round of node addition.

**Fig. 2:**
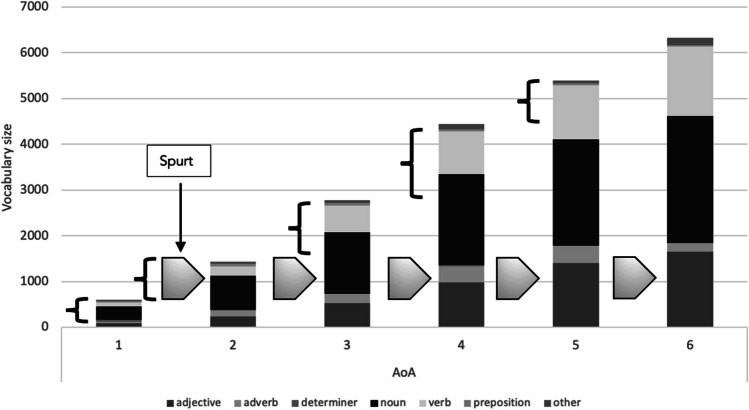
Schematization of growth spurts prior to and inbetween age of acquisiton (AoA) stages. The vocabulary sizes of each AoA stage with part-of-speech composition are indicated. Phonological neighborhood growth was tracked from a word’s initial entry to the lexicon until the most advanced acquisition stage, as schematized with arrows for words acquired at AoA stage 2. The point of entry (AoA stage) determines the number of growth spurts a word can undergo (i.e., five in the case of words learned at AoA stage 2)

For each word at each AoA, the group of new phonological neighbors gained to reach the next AoA stage was defined as the growth spurt for that particular target word. Starting at level AoA 0, words could go through six growth spurts: From 0 to 1, from 1 to 2, from 2 to 3, from 3 to 4, from 4 to 5, and from 5 to 6. Words acquired at later AoA stages have fewer options to expand their neighborhoods. Figure 2 schematizes the growth spurts as conceived for the present study.

In the final dataset, only words that experienced growth at some point of network evolution were included. While the majority of early learned words would grow neighbors during future rounds of word learning, the majority of late-acquired words would remain lexical hermits without neighbors. Previous research has shown that the L1-American English phonological network consists of 53% lexical hermits (see Vitevitch, 2008); the second-language phonological networks range between 27% and 66%. Table 3 presents the overall and final vocabulary sizes for all AoA stages.

**Table 3:** Vocabularies of the six age of acquisition stages

	1	2	3	4	5	6
Vocabulary size	584	1438	2783	4440	5386	6322
Unique words acquired at stage	584	899	1345	1657	946	936
Words experiencing growth	426	528	711	723	318	
Non-growing words (% of uniquely acquired words per stage)	27%	41%	47%	56%	66%	

All growing words of all AoA stages were copied into a spreadsheet containing the following variables: word, AoA (1–6), spurt number (1–6), lexical frequency rate (log), phonological length, L1-neighborhood density, in addition to the three dependent variables which are the focus of the present study: degree, saturation, and weighted degree. They are explained below.

### Growth spurt size analysis

Degree refers to the number of neighbors a node has in a network and thus equals neighborhood density in psycholinguistic terms. Degree accumulation per word was calculated as percentage increases from one level to the next. The total number of neighbors gained at the highest proficiency level AoA stage 6 was used to calculate the percentage-wise degree growth spurts at the lower levels. For instance, the word “bad” reaches a maximum of 16 neighbors at AoA stage 6; the gain of neighbors at the first AoA stage is 8 and thus 50% of the overall degree that the word will reach; at the second AoA stage three more neighbors are added, which brings the total proportion of neighbors known at this level to 69% and the overall growth difference between the AoA stages 1 and 2 to 19%.

The maximum number of possible growth spurts per word ranged between 2 and 6, depending on the AoA at which a word was acquired (cf. Fig. 2). Statistical growth spurt comparisons only make sense when there are at least two growth spurts left for a given AoA stage, thus the statistical models focus on growth spurts starting at AoA stages 1–5.

### Saturation analysis

In order to analyze how many word forms are available to English learners in relation to how many word forms they already know, a degree of neighborhood saturation was calculated. Saturation corresponds to the maximum number of phonological neighbors that are possible in L1-American English, and a saturation of level of 100% would indicate that all L1 neighbors have been learned at a specific L2 AoA stage. CLEARPOND was used to determine the number of L1 neighbors of target words, and that value was used as the 100% saturation benchmark. For each word at each AoA stage, a neighborhood saturation value was calculated by dividing the number of neighbors by the overall number of neighbors as listed in CLEARPOND and multiplying it by 100. For instance, the word “any” has 15 neighbors in L1, but only reaches a maximum of five words at the last AoA stage 6 in second-language learners. This equals a maximum saturation of 33% in ESL. The word has two neighbors at AoA stage 1, which equals a saturation proportion of 13%; at the next growth spurt (AoA 1 to 2) “any” does not gain new neighbors and saturation remains at 13%; but the word gains two more neighbors at the next growth spurt (AoA 2 to 3), which brings its saturation level to 26%. The proportional gain in saturation between AoA stages was calculated in the same way as the growth spurt size analysis.

### Phonological distance analysis

Weighted degree is a measure of the edge strengths within a node’s neighborhood. In the present study, weighted degrees were calculated as average sums of phonological distances between the phonological neighbors. Since weighted degree and degree centralities are correlated (in the sense that new neighbors also add weight), the average weighted degree of new neighbors acquired at the various growth spurts were calculated. For instance, the word “be” acquired at the first AoA stage starts out with seven neighbors and does not acquire any during the next growth spurt (AoA stage 1 to 2), and for both of those AoA stages the weighted degree equals 352. This results in an average weighted degree of 352/7 = 50.3 per neighbor at those AoA stages. After the next growth spurt (AoA 2 to 3), the neighborhood expands to 12 neighbors and weighted degree equals 704, resulting in an average weighted degree of 58.7 per new neighbor. Comparing the weighted degree fluctuations for new neighbors added at various stages of network development can inform about the timeline of when neighbors of certain phonetic proximity are added to the lexicon.

### Statistical analyses

To test the relationship between the proportional dependent variables (degree gain, saturation gain) and the independent variables AoA (1–6), growth spurt (1–6), frequency (log-transformed), length (number of phonemes), a generalized linear model with *quasi-binomial logit-link* function was employed. The proportional variables were transformed to range within 0 and 1 (avoiding the extreme values) following Smithson & Verkuilen’s (2006) suggestion with the equation:

$$\left(\mathrm{y}*\left(\mathrm{n}-1\right)+0.5\right)/\mathrm{n}$$

with *y* being the proportional variable and *n* the sample size.

Two models were constructed with the proportional dependent variables. The following pseudo code shows the model structure:

$$\mathrm{Proportional}\;\mathrm{DV}\sim\mathrm{Spurt}+\mathrm{AoA}+\mathrm{Frequency}+\mathrm{Length},\mathrm{family}=\mathrm{quasibinomial}\;(\mathrm{link}=\mathrm{logit})$$

Lexical frequency rate (“Frequency”) and length of words (“Length”) were coded as continuous numeric variables. Age of acquisition (AoA) and Spurt were assigned categorical ordinal structures in the analyses. With the *factor* function in R, the levels and their ascending orders were set. Words were nested within proficiency levels, and each word was assigned to only one AoA (i.e., the AoA stages at which a word is added to the lexicon).

Due to issues with overdispersion in glm models and a large number of zero values in the data, the weighted degree model was constructed as a zero-inflated negative binomial regression (Long, 1997; Zuur & Ieno, 2016) but following a similar pattern as the previous models:

$$\mathrm{DV}\sim \mathrm{Spurt}+\mathrm{AoA}+\mathrm{Frequency}+\mathrm{Length},\mathrm{ dist}=\mathrm{^{\prime}}\mathrm{negbin{^\prime}}$$

Since it is known that early-acquired words tend to differ in lexical characteristics from those acquired at more advanced learning stages, which also applies to phonological networks (e.g., Siew, 2013; Stadthagen-Gonzalez et al., 2004), an overview of how “old” and “new” words differ in a number of variables will be presented. Certain growth patterns will also be analyzed to see how they relate to lexical characteristics of words. In addition, each network will be inspected for degree assortativity to understand the affiliation patterns between words with dense and those with sparse neighborhoods.

The sample size of the statistical models was 10,845 words. The proportional models were implemented in R (R Core Team, 2021) using the function *glm* of the package ‘lme4’. The zero-inflated model was implemented in ‘pscl’ with the function *zeroinfl*. Assumption diagnostics were performed with the packages ‘car’, ‘performance’, ‘countreg’, and ‘DHARMa’. The package ‘lsmeans’ was used for post hoc tests of the spurts. Effect size metrics were calculated with the package ‘effectsize’. MacFadden R-squared values were obtained with the following formula: 1–(log likelihoodmodel /log likelihoodnull). Network statistics were calculated with ‘igraph’. For visualizations and plot statistics, ‘ggpubr’ and ‘ggplot2’ were used.

## Results

### Growth spurt size analysis

Results indicated that AoA and Spurt showed various significances, while frequency and length had no effect on proportional degree gain in the model (see Table 4).

**Table 4:** Proportional degree gain

	Estimate	Std. error	t value	Pr(>|z|)
(Intercept)	9.765e-02	1.120e-01	0.872	0.38
AoA 2	1.075e-01	6.361e-02	1.690	0.09
AoA 3	2.296e-01	6.703e-02	3.426	< 0.001 ***
AoA 4	4.400e-01	7.425e-02	5.926	3.2e-09 ***
AoA 5	8.563e-01	9.605e-02	8.915	< 2e-16 ***
Spurt 2	-1.908e+00	4.642e-02	-41.104	< 2e-16 ***
Spurt 3	-2.151e+00	5.214e-02	-41.259	< 2e-16 ***
Spurt 4	-2.427e+00	6.747e-02	-35.966	< 2e-16 ***
Spurt 5	-2.763e+00	1.003e-01	-27.539	< 2e-16 ***
Spurt 6	-2.724e+00	1.491e-01	-18.272	< 2e-16 ***
Frequency (log)	1.173e-11	3.296e-02	0.0	1.0
Length	6.694e-11	1.122e-02	0.0	1.0

< 0.001 = ***, < 0.01 = **, > 0.05 = *

McFadden’s R-squared = 0.3

There were no signs of overdispersion in the model (residual deviance was 5,632 on 10,833 degrees of freedom; dispersion parameter = 0.53); all variance inflation factors of the variables were below 2.3. Odds ratios of all variables and their interpretations are reported in Table 5. The variable Spurt can be seen to have the largest effect in the model.

**Table 5:** Odds ratios

	Standardized OR	Interpretation of effect size (Cohen, 1988)
(Intercept)	1.1	Very small
AoA 2	1.11	Very small
AoA 3	1.26	Very small
AoA 4	1.55	Small
AoA 5	2.35	Small
Spurt 2	0.15	Large
Spurt 3	0.12	Large
Spurt 4	0.09	Large
Spurt 5	0.06	Large
Spurt 6	0.07	Large
Frequency (log)	1.0	Very small
Length	1.0	Very small

*AoA* age of acquisition

**Fig. 3:**
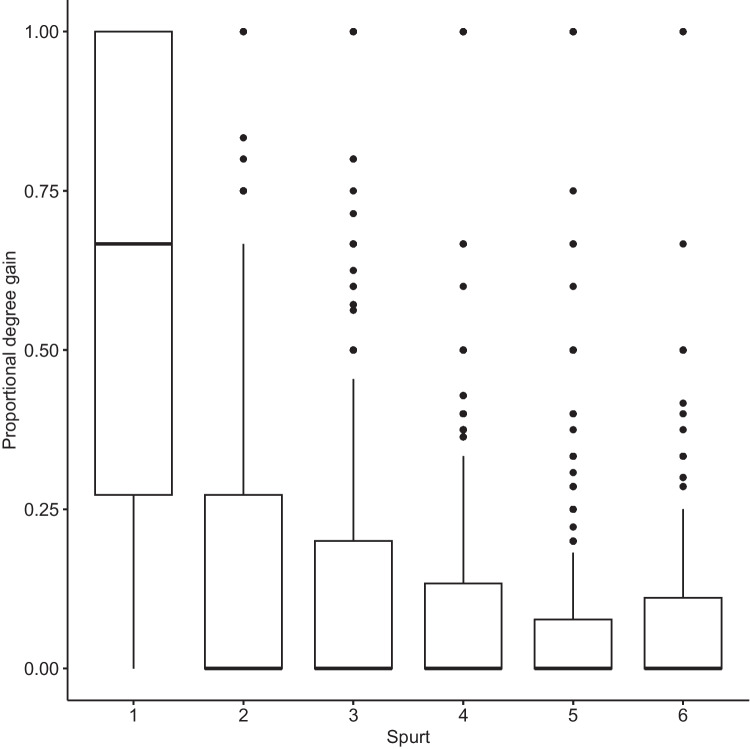
Proportional degree gain in growth spurts. The first growth spurt adds the large majority of new words. Consecutive ones are markedly smaller in size

The proportion of degree gain decreases with later growth spurts. Figure 3 illustrates the dominance of the first growth spurt.

**Fig. 4:**
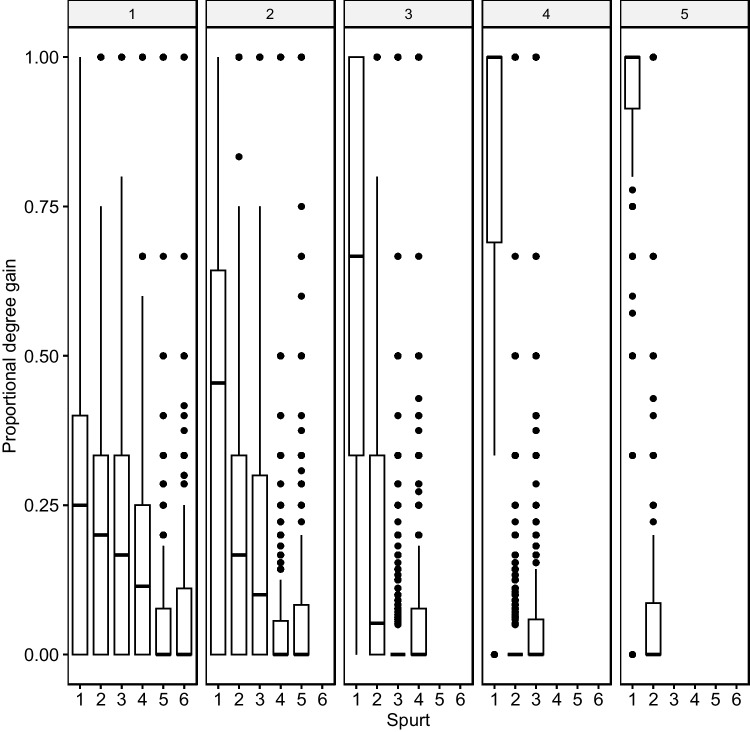
Growth spurts and word age of acquisition (AoA) stages. Facets indicate word age (e.g., 1 = AoA stage 1). The first growth spurt is the largest at all AoA stages, the later ones are smaller. There is a seeming exacerbation of this trend at more advanced AoA stages. Late-acquired words undergo fewer growth spurts

The first growth spurt was always the largest for all words acquired at all AoA stages, with later growth spurts smaller in scope (see Fig. 4). Words undergo their largest growth spurt when they are new to the lexical system but show shrinking growth as they age. A comparison of the growth decline from the first to the second spurt reveals a steadily growing gulf between first and second spurts that reaches quite pronounced degrees at more advanced AoA stages, where the large majority of neighborhood growth occurs during the first growth spurt. Words acquired at the more advanced AoA stages experience growth almost exclusively in the initial spurt.

As post hoc analysis, estimated proportions were obtained from the model (using the function *type = ”response”*) in the package ‘lsmeans’. The pairwise comparisons of the results (Tukey’s method) were calculated via odds ratios, which were then re-gridded to the response scale (with the function *pairs(regrid))* to get differences in proportions. The results of the pairwise comparisons of growth spurts indicate significant size differences between the majority of spurts (see Table 6).

**Table 6:** Results of the pairwise comparisons (Tukey’s method, results averaged over age of acquisition (AoA) stages)

Comparison	Estimate	Std. error	z ratio	p
Spurts 1 – 2	0.41968	0.00885	47.396	<.0001 ***
Spurts 1 – 3	0.45351	0.00880	51.518	<.0001 ***
Spurts 1 – 4	0.48555	0.00940	51.665	<.0001 ***
Spurts 1 – 5	0.51658	0.01043	49.521	<.0001 ***
Spurts 1 – 6	0.51337	0.01405	36.550	<.0001 ***
Spurts 2 – 3	0.03384	0.00787	4.301	<.0002 ***
Spurts 2 – 4	0.06587	0.00852	7.734	<.0001 ***
Spurts 2 – 5	0.09691	0.00958	10.119	<.0001 ***
Spurts 2 – 6	0.09369	0.01342	6.983	<.0001 ***
Spurts 3 – 4	0.03203	0.00848	3.776	0.002 **
Spurts 3 – 5	0.06307	0.00958	6.581	<.0001 ***
Spurts 3 – 6	0.05986	0.01341	4.463	0.0001 **
Spurts 4 – 5	0.03104	0.00988	3.142	0.021 *
Spurts 4 – 6	0.02782	0.01361	2.043	0.32
Spurts 5 – 6	-0.00321	0.01409	-0.228	0.99

Significance: < 0.001 = ***, < 0.01 = ** < 0.05 = *

To test whether high-degree nodes preferentially acquire high-degree neighbors, Pearson correlation coefficients were calculated between the degrees of neighboring nodes (Newman, 2002). Using the *assortativity.degree* function in the R package ‘igraph’, medium and positive correlations between node degrees in all six networks were revealed (see Table 7).

**Table 7:** Assortativity by degree in the six networks

AoA	1	2	3	4	5	6
*Pearson r*	0.53	0.49	0.58	0.6	0.52	0.41

*AoA* age of acquisition

**Fig. 5:**
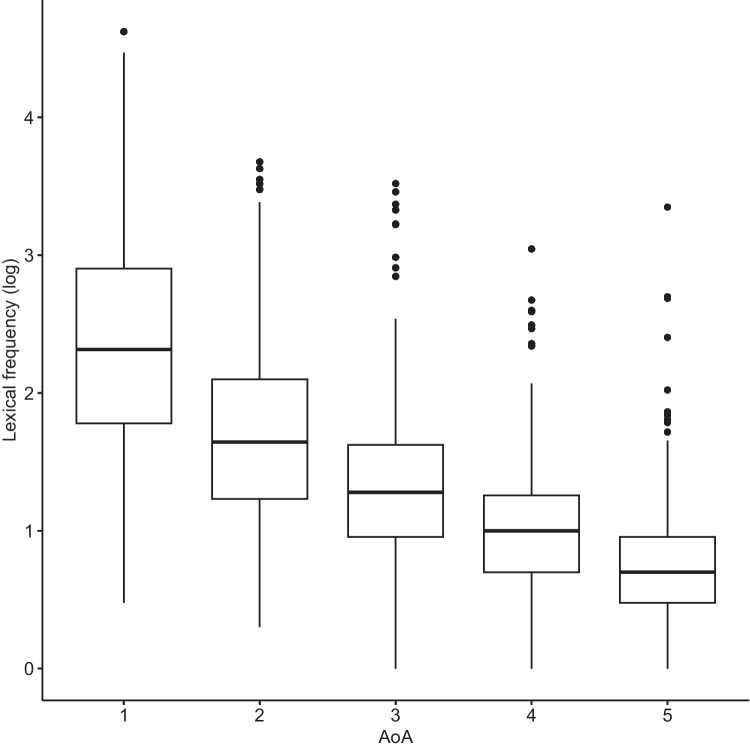
Lexical frequency rates of words added at various age of acquisition (AoA) stages. Words acquired at earlier AoA stages were generally of higher frequency

**Fig. 6:**
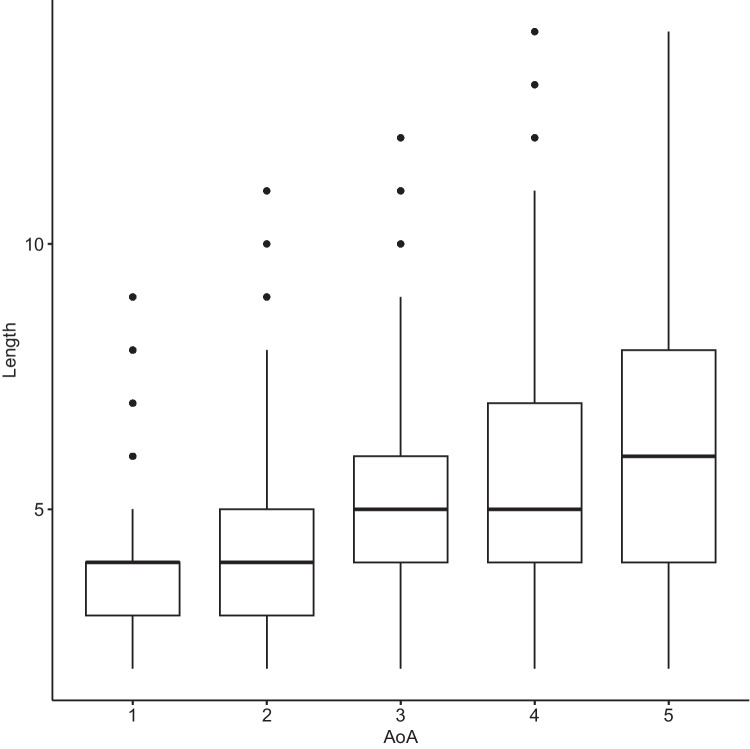
Phonemic length of words added at various age of acquisition (AoA) stages. Words acquired at earlier AoA stages were generally shorter

**Fig. 7:**
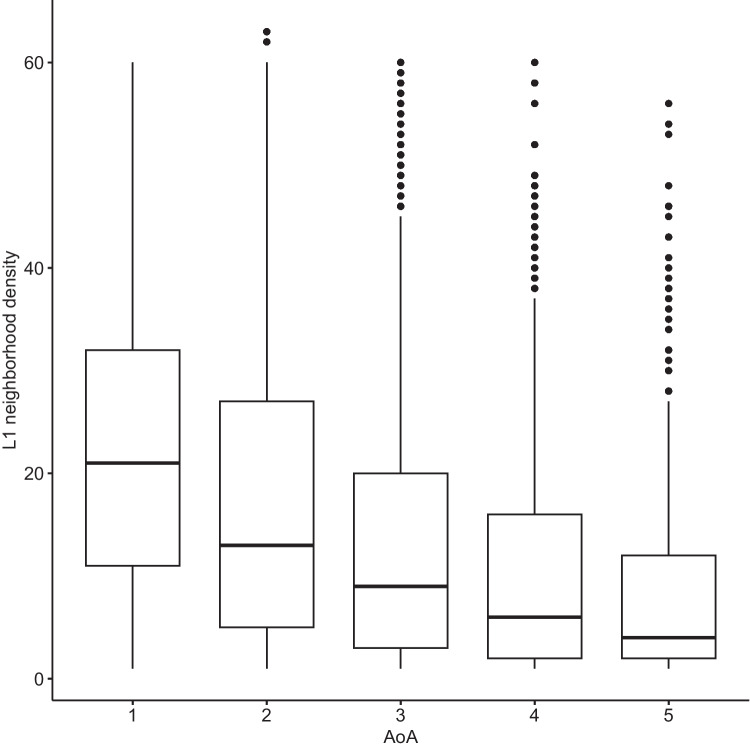
L1-phonological neighborhood density of words added at various age of acquisition (AoA) stages. Words acquired at earlier AoA stages had denser L1 neighborhoods

Lexical frequency rates were shown to be higher while words were generally shorter in earlier AoA stages (see Figs. 5, 6 and 7).

### Saturation

On average, saturation levels were quite low (see Table 8), with the most advanced AoA stage reaching only 50% of the L1-given neighborhood density.

**Table 8:** Average saturation achieved for words acquired at different age of acquisition (AoA) stages (proportion of L1-given neighborhood density)

A1	A2	B1	B2	C1
33.4% (St.dev.=15)	37.3% (St.dev.=22)	40.7% (St.dev.=24.4)	45.3% (St.dev.=27.1)	50.9% (St.dev.=30.9)

Statistical results indicate various significances with regards to proportional saturation gains across AoA stages and spurts (see Table 9).

**Table 9:** Proportional saturation gain

	Estimate	Std. error	t value	Pr(>|z|)
(Intercept)	-1.892309	0.112691	-16.792	< 2e-16 ***
AoA 2	0.131364	0.072142	1.821	0.0686
AoA 3	1.151011	0.070310	16.370	< 2e-16 ***
AoA 4	0.378343	0.078739	4.805	1.57e-06 ***
AoA 5	0.647122	0.093567	6.916	4.90e-12 ***
Spurt 2	-1.879545	0.046455	-40.460	< 2e-16 ***
Spurt 3	-2.271981	0.056602	-40.140	< 2e-16 ***
Spurt 4	-2.792649	0.080328	-34.766	< 2e-16 ***
Spurt 5	-2.614874	0.129727	-20.157	< 2e-16 ***
Spurt 6	-2.397366	0.186334	-12.866	< 2e-16 ***
Frequency (log)	0.009405	0.033838	0.278	0.7811
Length	0.140519	0.010146	13.849	< 2e-16 ***

< 0.001 = ***

McFadden’s R-squared = 0.4

*AoA* age of acquisition

The model showed no signs of overdispersion (dispersion parameter = 0.31) and all variance inflation factors of the variables were below 2.1. Standardized odds ratios can be found in Table 10. As in the degree gain model, the variable Spurt had the largest effect in this model.

**Table 10:** Odds ratios

	Standardized OR	Interpretation of effect size (Cohen, 1988)
(Intercept)	0.3	Medium
AoA 2	1.14	Very small
AoA 3	3.16	Medium
AoA 4	1.46	Small
AoA 5	1.91	Small
Spurt 2	0.15	Large
Spurt 3	0.1	Large
Spurt 4	0.06	Large
Spurt 5	0.07	Large
Spurt 6	0.09	Large
Frequency (log)	1.01	Very small
Length	1.29	Very small

*AoA *age of acquisition

**Fig. 8:**
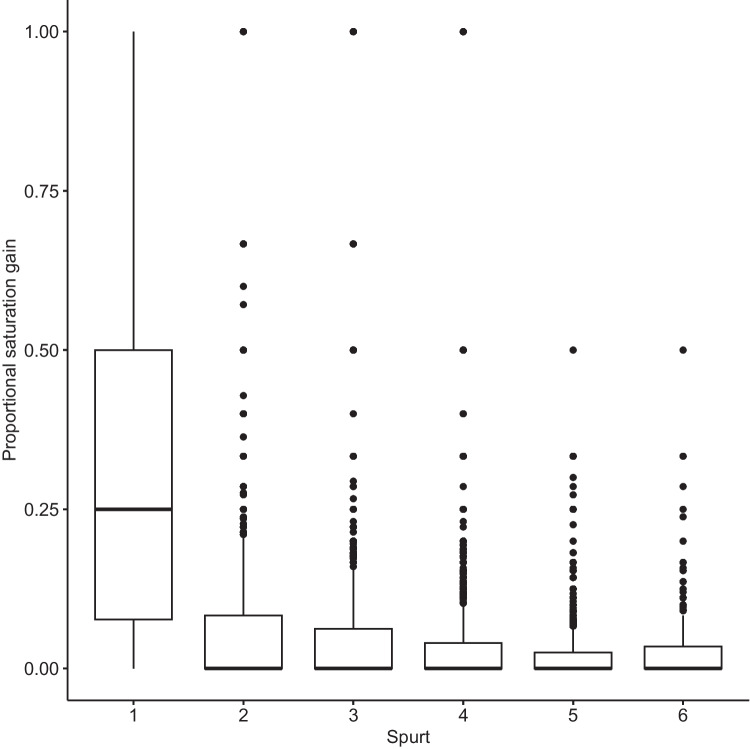
Proportional saturation gain in growth spurts. The first growth spurt adds the largest saturation gain to phonological neighborhoods

The first growth spurt added the largest saturation to phonological neighborhoods (see Fig. 8).

**Fig. 9:**
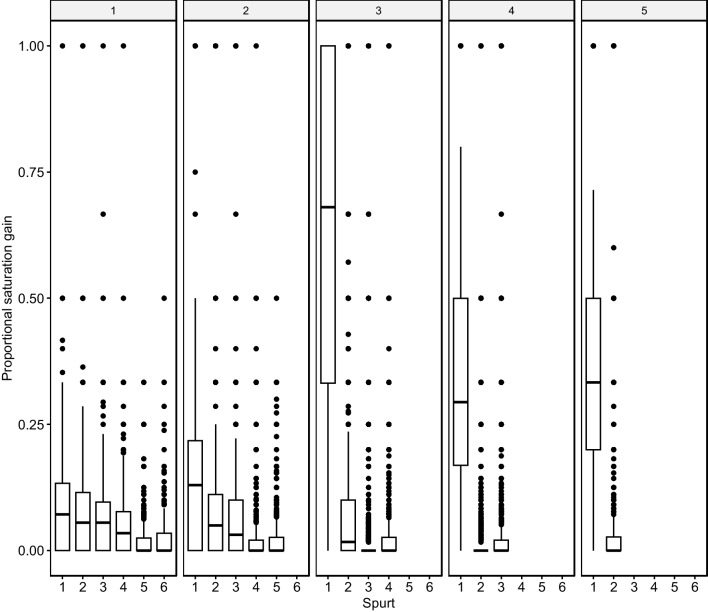
Saturation spurts and age of acquisition (AoA). Facets indicate word age (e.g., 1 = AoA stage 1). The first saturation spurt is the largest at all AoA levels, later ones are smaller. There is a seeming exacerbation of this trend at higher AoA stages. Late-acquired words undergo fewer growth/ saturation spurts

Figure 9 shows the saturation rates per AoA stage. The first spurt is the most significant saturation spurt in more advanced lexica.

Pairwise comparisons of spurts (regridded as described above) indicated various significant differences between the first and second spurts compared to the rest of the spurts (see Table 11).

**Table 11:** Results of the pairwise comparisons (Tukey’s method, results averaged over age of acquisition (AoA) stages)

Comparison	Estimate	Std. error	z ratio	p
Spurts 1 – 2	0.25469	0.00581	43.872	<.0001 ***
Spurts 1 – 3	0.27565	0.00570	48.338	<.0001 ***
Spurts 1 – 4	0.29409	0.00572	51.440	<.0001 ***
Spurts 1 – 5	0.28877	0.00687	42.048	<.0001 ***
Spurts 1 – 6	0.28093	0.00924	30.402	<.0001 ***
Spurts 2 – 3	0.02095	0.00340	6.170	<.0001 ***
Spurts 2 – 4	0.03940	0.00329	11.963	<.0001 ***
Spurts 2 – 5	0.03407	0.00494	6.892	<.0001 ***
Spurts 2 – 6	0.02624	0.00784	3.345	0.011
Spurts 3 – 4	0.01844	0.00309	5.978	<.0001 ***
Spurts 3 – 5	0.01312	0.00478	2.746	0.07
Spurts 3 – 6	0.00528	0.00772	0.684	0.98
Spurts 4 – 5	-0.00533	0.00464	-1.148	0.86
Spurts 4 – 6	-0.01316	0.00763	-1.725	0.51
Spurts 5 – 6	-0.00784	0.00831	-0.943	0.94

< 0.001 = ***

### Phonetic distance analysis: Weighted degree

The majority of average weighted degree centralities ranged between 50 and close to 100, with a sizable portion of zeros reflecting the fact that some initial growth spurts did not add any neighbors (i.e., delayed growth, this will be discussed below). Results showed some significant differences in phonetic distances between neighbors per growth spurt (see Table 12).

**Table 12:** Phonetic distances

Count model coefficents (negbin with log link):				
	Estimate	Std. error	z value	Pr(>|z|)
(Intercept)	4.2906288	0.0069289	619.235	<2e-16 ***
AoA 2	0.0032583	0.0037054	0.879	0.3792
AoA 3	0.0065999	0.0040709	1.621	0.1050
AoA 4	0.0006625	0.0046498	0.142	0.8867
AoA 5	-0.0126675	0.0062836	-2.016	0.0438 *
Spurt 2	0.0008026	0.0032555	0.247	0.8053
Spurt 3	0.0046358	0.0033778	1.372	0.1699
Spurt 4	0.0047173	0.0038367	1.230	0.2189
Spurt 5	0.0116063	0.0047060	2.466	0.0137 *
Spurt 6	0.0141635	0.0065454	2.164	0.0305 *
Frequency (log)	-0.0209136	0.0019393	-10.784	<2e-16 ***
Length	0.0281118	0.0006986	40.241	<2e-16 ***
Log (theta)	17.5891181	NaN	NaN	NaN
Zero-inflation model coefficients (binomial with logit link):				
	Estimate	Std. error	z value	Pr(>|z|)
(Intercept)	-1.75000	0.22407	-7.810	5.72e-15 ***
AoA 2	-0.83497	0.11886	-7.025	2.14e-12 ***
AoA 3	-1.67236	0.13222	-12.648	< 2e-16 ***
AoA 4	-2.95065	0.16801	-17.562	< 2e-16 ***
AoA 5	-4.31933	0.27910	-15.476	< 2e-16 ***
Spurt 2	-1.13819	0.09118	-12.483	< 2e-16 ***
Spurt 3	-2.41878	0.13594	-17.793	< 2e-16 ***
Spurt 4	-3.70336	0.24670	-15.012	< 2e-16 ***
Spurt 5	-5.12786	0.58356	-8.787	< 2e-16 ***
Spurt 6	-17.02084	305.54934	-0.056	0.956
Frequency (log)	-0.30390	0.07232	-4.202	2.64e-05 ***
Length	0.45444	0.02377	19.115	< 2e-16 ***

< 0.001 = ***, > 0.05 = *

*AoA* age of acquisition

Dispersion did not appear to be an issue in the model (dispersion parameter = 0.9). Effect sizes are reflected in the standardized odds ratios in see Table 13.

**Table 13:** Odds ratios

Component		Standardized OR	Interpretation of effect size (Cohen, 1988)
Conditional	Count_(Intercept)	80.87	Large
	AoA 2	1.0	Very small
	AoA 3	1.01	Very small
	AoA 4	1.0	Very small
	AoA 5	0.99	Very small
	Spurt 2	1.0	Very small
	Spurt 3	1.0	Very small
	Spurt 4	1.0	Very small
	Spurt 5	1.37	Very small
	Spurt 6	1.01	Very small
	Frequency (log)	1.01	Very small
	Length	0.98	Very small
Zero-inflated	Zero_(Intercept)	0.96	Very small
	AoA 2	0.43	Small
	AoA 3	0.19	Large
	AoA 4	0.05	Large
	AoA 5	0.01	Large
	Spurt 2	0.32	Medium
	Spurt 3	0.09	Large
	Spurt 4	0.02	Large
	Spurt 5	5.93e-03	Large
	Spurt 6	4.05e-08	Large
	Frequency (log)	0.78	Very small
	Length	2.26	Small

*AoA* age of acquisition

The pairwise comparisons of spurts indicated numerous significances (see Table 14).

**Table 14:** Results of the pairwise comparisons (Tukey’s method, results averaged over age of acquisition (AoA) stages)

Comparison	Estimate	Std. error	z ratio	p
Spurts 1 – 2	-9.270	0.733	-12.643	<.0001 ***
Spurts 1 – 3	-14.285	0.673	-21.217	<.0001 ***
Spurts 1 – 4	-15.858	0.668	-23.722	<.0001 ***
Spurts 1 – 5	-16.900	0.697	-24.245	<.0001 ***
Spurts 1 – 6	-17.265	0.784	-22.014	<.0001 ***
Spurts 2 – 3	-5.015	0.562	-8.932	<.0001 ***
Spurts 2 – 4	-6.589	0.553	-11.918	<.0001 ***
Spurts 2 – 5	-7.630	0.588	-12.980	<.0001 ***
Spurts 2 – 6	-7.995	0.690	-11.589	<.0001 ***
Spurts 3 – 4	-1.573	0.422	-3.729	0.003 **
Spurts 3 – 5	-2.615	0.464	-5.632	<.0001 ***
Spurts 3 – 6	-2.980	0.589	-5.061	<.0001 ***
Spurts 4 – 5	-1.041	0.422	-2.467	0.13
Spurts 4 – 6	-1.407	0.556	-2.530	0.12
Spurts 5 – 6	-0.365	0.568	-0.644	0.98

< 0.001 = ***, < 0.01 = **

**Fig. 10:**
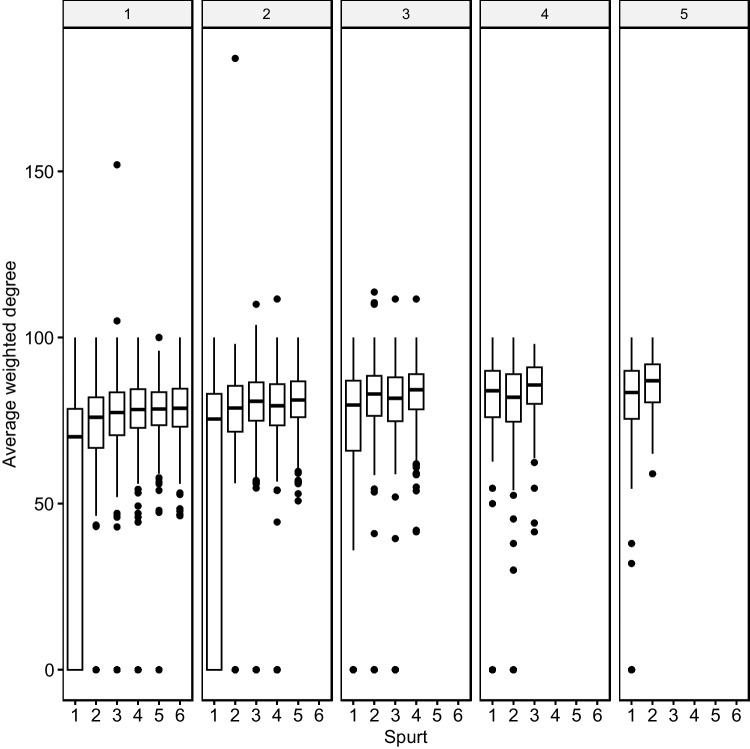
Phonetic distances in neighbors. Facets indicate word age (e.g., 1 = age of acquisition (AoA) stage 1). Average phonetic distances (i.e., edge weights) per growth spurts and at the different stages of AoA. Closer phonetic neighbors are more likely to be added to the lexicon at more advanced stages. Late-acquired words undergo fewer growth spurts

The earliest growth spurts seem to add more phonetically varied neighbors (close, medium, distant), while later spurts add primarily phonetically closer neighbors. Figure 10 illustrates this neighborhood growth trend.

Words acquired at earlier AoA stages are characterized by greater phonetic distances (mean weighted degree: *AoA*- stage 1 = 70.4, stage 2 = 73.2, stage 3 = 75.6, stage 4 = 79.2, stage 5 = 81.6). At AoA stages 1 and 2, there is a significant number of zero growth, indicating that growth patterns differ between AoA stages.

### Growth patterns

The majority of growth patterns was quite varied in the L2 phonological networks, but the following distinct growth patterns could be discerned that can provide insights into the timing of growth in relation to AoA stages:

- *Initial growth*: a word is introduced in the lexical system but does not undergo growth during any of the following growth spurts

- X+0+0…

- *Continuous growth*: after its introduction, a word continues to grow during all spurts

- X+1+1…

- *Delayed growth*: instances where a new word lays dormant for the first or first and second spurts and only undergoes growth at later spurts

- X+0+1+1…
- X+0+0+1…

**Fig. 11:**
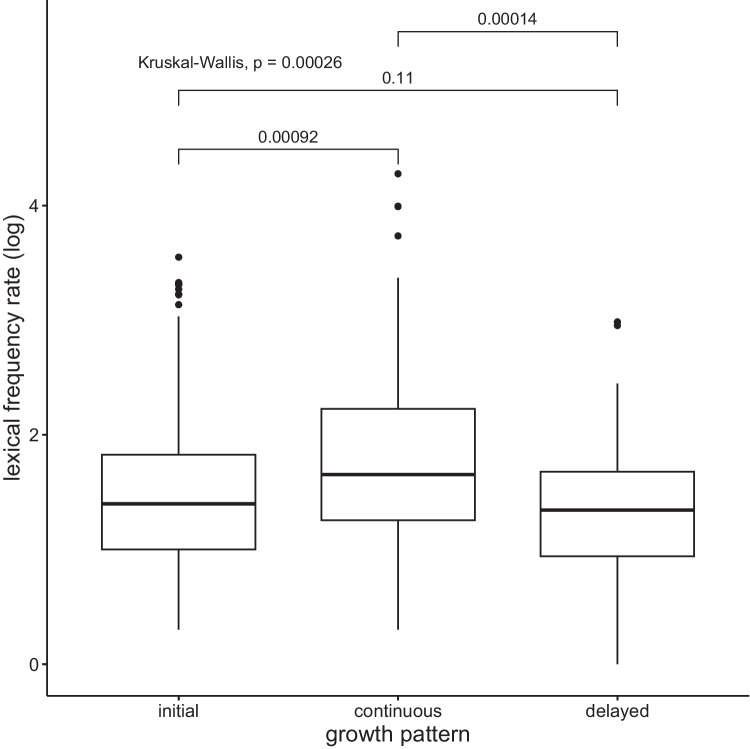
Growth patterns and lexical frequency rate. Wilcoxon pairwise comparisons revealed that higher lexical frequency rate was a factor in continuous lexical growth

This analysis concerns only a subset of the data extracted from AoA stages 1, 2, and 3, as words acquired at these levels have the opportunity to experience multiple growth spurts after their introduction to the lexicon. A total of 512 words were identified to grow according to the three patterns. Of these, 319 words grew initially, 101 words grew continuously, and 93 words grew in a delayed manner. Higher-frequency words had a better chance of growing continuously through all growth spurts (see Fig. 11).

**Fig. 12:**
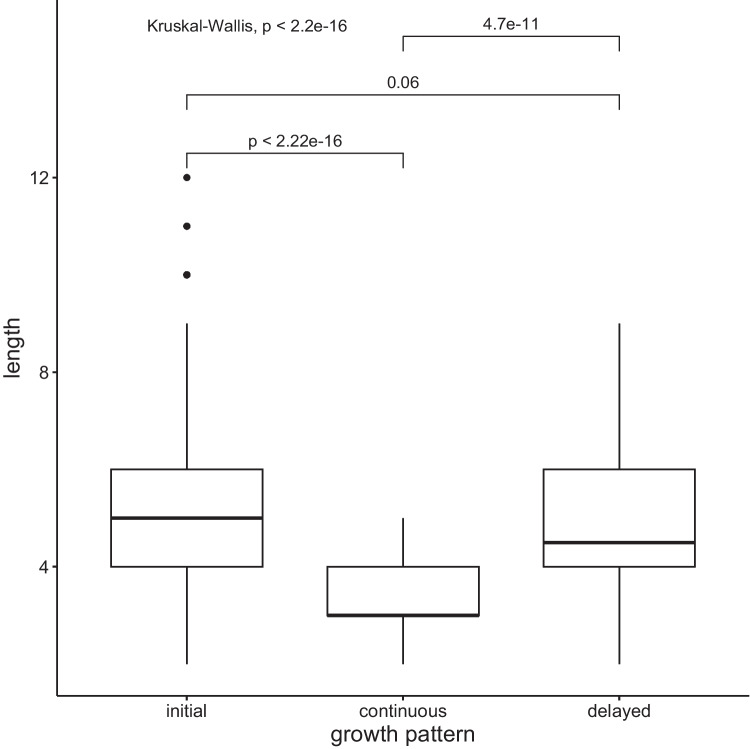
Growth patterns and phonemic length. Wilcoxon pairwise comparisons revealed that shorter words were more likely to grow continuously

At the same time, words that displayed continuous growth were also of shorter phonemic length (see Fig. 12).

**Fig. 13:**
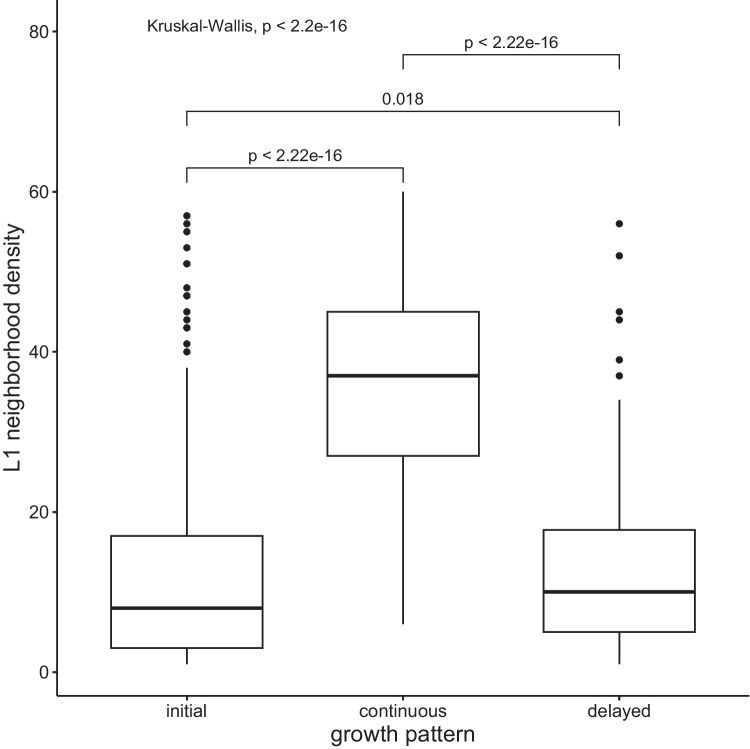
Growth patterns and L1 neighborhood density. Wilcoxon pairwise comparisons revealed that words with denser neighborhoods in L1 were more likely to grow continuously

Continuously growing words were also characterized by a denser neighborhood in L1-American English (see Fig. 13).

These results likely reflect the fact that denser neighborhoods tend to be composed of shorter and more frequent words, a well-known phonological neighborhood statistic (e.g., Pisoni et al., 1985; Vitevitch & Rodriguez, 2004; Zipf, 1935).

## Discussion

This study investigated obsolescence effects in degree and weighted degree growth of a preferential attachment-driven phonological network of second-language learners of English. Results demonstrate that words realize their largest growth potential initially but lose their growth relevance over time. This pattern was also evident in L1-neighborhood exhaustion, and words realize their largest L1-saturation at earlier growth spurts. In addition, it was shown that the earlier AoA stages add phonetically more distant phonological neighbors. Overall, the findings demonstrate obsolescence effects in network growth and saturation that mitigate preferential attachment.

Phonological networks are classified as preferential attachment network models (Vitevitch, 2008); however, pure preferential attachment is not sufficient to describe the growth process in those networks (Vitevitch, 2021). An obsolescence effect of node relevance in relation to a node’s age in the network can explain some of the observed growth dynamics. Obsolescence benefits younger nodes, which have only recently been introduced to the lexical system. With each spurt, growth steadily declines, and this gap between the first and consecutive growth spurts widens with increasing language proficiency. At the most evolved stages of the L2 network, the obsolescence effect seems most pronounced, with the youngest nodes capturing the overwhelming majority of new incoming links, whereas older nodes have clearly compromised their ability to obtain new neighbors. A preferential-attachment-induced first-mover effect was absent from the second-language phonological network, but the temporal growth effect was primarily focused on younger nodes, establishing a “late-mover effect” where being new draws more future neighbors (Barabási, 2016; Dorogovtsev & Mendes, 2000).

The findings indicate phonological neighborhood construction where the majority of neighborhood growth takes places in one large initial acquisition spurt, followed by near-dormancy of these neighborhoods afterwards. The bulky acquisition of similar word forms is known as “leveraged learning” or lexical bursts in language acquisition research (Mitchell & McMurray, 2009). It entails the assumption that words have intrinsic qualities that impact their learnability, and words of these same qualities (e.g., phonology, phonotactics) are acquired together (Elman et al., 1996; Mitchell & McMurray, 2008; van Geert, 1991). As stated by Mitchell & McMurray (2009, p. 1), “knowledge of some words helps with the learning of others.” Leveraged learning focusses the learner’s attention on a small range of phonological segments and phonotactic possibilities, ultimately facilitating the acquisition-by-similarity principle operating on word form learning. While lexical bursts are primarily known from first-language acquisition (e.g., Albin & Gershkoff-Stowe, 2016; Bloom, 2000; Ganger & Brent, 2004), their existence in second languages is suggested by preferential attachment growth in the development of phonological networks of second-language learners (Luef, 2022a).

Preferential attachment presents a probabilistic conundrum in phonological networks. It is well known that low-degree nodes are more abundant, while high-degree nodes are significantly scarcer in first and second languages (Arbesman et al., 2010; Luef, 2022a; Vitevitch, 2008). Thus, it should be more likely that growth involves low-degree nodes. The fact that this has not been observed in phonological networks points to node relevance as the driving force behind growth. Rather than act in a purely probabilistic way, human lexical cognition seems to be geared toward increasing efficiency and making best use of the phonological resources already present in the lexicon. By focusing on leveraged learning, large parts of the lexicon become neglected in terms of their growth potential; however, at the benefit of the small part of the lexicon that can maximize growth. This small growing part may be similar to what has been described as the “backbone” of a network (Vitevitch & Sale, 2023), where the extraction of the core of a phonological lexicon can yield insights into the basic scaffolding of phonological word form composition in a language.

What the present results clearly show is that not much effort goes into attaching new neighbors to old neighborhoods. The early learned words are characterized by shorter length, higher phonotactic probability, and higher lexical frequency rate, and generally have denser L1 neighborhoods (also see Siew, 2013), yet the first-spurt acquisition dynamic is also very prominent in later stages of network development, indicating that even low-frequency, longer words with fewer neighbors tend to be acquired in one large spurt. This may explain the PATT-iPATT switch in phonological networks: the switch may result from the obsolescence effect of the network combined with the fact that late-arriving words are generally of lower degree. At each AoA stage, the first growth spurt is the largest, and this also applies to stage 5. It is just that the words introduced at the later stages are of lower neighborhood density in general, leading to the iPATT effect in network growth. Central tenets of the iPATT-hypotheses are confirmed by the present study: words acquired at later AoA stages tend to be of sparser neighborhood density (in L1 and L2) and there is a certain degree of neighborhood saturation, after which learners stop expanding neighborhoods and re-directed growth in their lexicon.

The saturation analysis demonstrates that L2 neighborhoods reach a density ceiling without having exhausted all possible neighbor options in a lexicon. L2 learners may invest fewer cognitive resources into bulky lexical acquisition, yielding a ceiling effect where neighborhood density remains limited even in highly advanced learners of a second language. Smaller initial acquisition spurts coupled with the obsolescence effect that prevents new words from becoming attached to old neighborhoods could lead to the observed neighborhood density limitations.

In general, the low L2 neighborhood saturation demonstrates a crucial difference in neighborhood density between L1 and L2, and carries implications for phonological neighborhood metrics in L2. The low saturation rates also demonstrate that L2 learners make selective use of the L1-given lexicon. As suggested by preferential attachment in developing phonological networks (Laing, 2022; Luef, 2022a; Siew & Vitevitch, 2020a), the ambient language environment (i.e., the fully-fledged adult first language) does not exert as much influence on the lexical development as would be expected. A limitation of the saturation analysis that has to be kept in mind is that early learned words (AoA stages 1 and 2) are characterized by shorter length, which means that they have more neighbors – and thus more saturation potential – in general. As the lexicon continues to be built up, words with sparser neighborhoods and less saturation potential become added, making it easier to achieve higher overall saturation levels in them as compared to the older words. For instance, the word “data” introduced at AoA stage 4 can have up to five neighbors, while the word “cat” learned at AoA stage 1 can have a maximum of 50 neighbors. The overall saturation rate of the “data” neighborhood reaches 80%, while saturation of the “cat” neighborhood remains at 34%. Saturation in low-density neighborhoods can generally reach higher proportions.

The phonological distance analysis should be treated with caution due to the low effect sizes. However, some observed trends may warrant further study. Beginning L2 learners seem to prefer phonetically distant neighbors, possibly due to their heightened phonological discriminability (similar to L1, see Storkel et al., 2006; Storkel & Rogers, 2000). Moreover, it seems that words acquired early are generally characterized by more phonetic distance.

The late-mover effect yielded for the L2 phonological network may have an influence on lexical processing. First-mover neighborhoods are more diverse in terms of node ages, as they incorporate words acquired in numerous acquisition spurts. It has been shown that the age of a word in a lexicon is predictive of how fast and reliably a word can be retrieved from memory, an effect potentially stemming from delayed retrieval of younger words (Brown & Watson, 1987; Navarrete et al., 2015; Perret et al., 2014). In addition, older word age is suggested to lower the activation threshold of word forms and cause more robust lexical memory (Alario et al., 2004; Gilhooly & Watson, 1981). Karimi & Diaz (2020) found that older words are activated more strongly, with consequences for lexical competition between words of different ages within a phonological neighborhood. According to their findings, same-age words have similarly strong activation levels and thus compete with one another more strongly than words of different ages. Subsequently, too many same-age words in a phonological neighborhood create a processing disadvantage, while neighborhoods consisting of mixed-age words are more beneficial. In a similar vein, phonological distances between phonological neighbors influence target word retrieval, and close neighbors act as stronger competitors, subsuming larger portions of the overall neighborhood activation, than more distant phonological neighbors (Mirman & Kittredge, 2010). Neighborhoods containing more distant neighbors could provide a processing advantage by reducing the interference stemming from neighborhood co-activation. However, these findings pertain to speech perception and retrieval, and opposite effects have been described for speech production. In the production domain, denser neighborhoods (i.e., those with more neighbors) may protect better against speech errors by strengthening production-relevant representations (Harley & Bown, 1998; Vitevitch, 1997, 2002; Vitevitch & Sommers, 2003) and resulting in more accurate and faster production rates of words embedded within dense neighborhoods (Vitevitch, 2002). The late-mover effect in the L2 phonological networks could suggest that bursty lexical learning in a second language leads to improved speech production rather than perception. Under this assumption, large acquisition lumps of phonologically similar words could bootstrap one another in a facilitated learning process that strengthens their production features. The data underlying the present study stems from a corpus that primarily contains spoken language, and the reported results should be interpreted accordingly.

A caveat of the present study concerns the determination of growth (and saturation) spurts that is dependent on the rather artificial classification of the proficiency levels of second languages according to the CEFR levels. This is similar to (even if potentially more problematic than) the age-of-acquisition norms that have been established for child language development (Brysbaert & Biemiller, 2016; Kuperman & Van Dyke, 2013). These proficiency classifications (in L1 and L2) rely on a specific lexical acquisition benchmark of the language learners and may miss a more gradual acquisition process that might be identifiable if one were to employ very fine-grained classifications of the lexical acquisition phases. In the absence of any other tools for defining language proficiency stages, these acquisition/proficiency norms are the most accurate measurements at researchers’ disposal and serve as the basis for language development analyses at present. However, in-depth case studies are able to look at lexical growth on a finer scale and may yield different patterns. Such future studies may contribute to a more detailed understanding of phonological network growth in L2 learners.

In sum, the results of the present study demonstrate an obsolescence effect in phonological neighborhood growth that goes hand in hand with a weakening of preferential attachment over the course of language learning. Node relevance declines with age in all evolutive stages of the second-language network.

## Data Availability

The data and R scripts that support the findings of this study are available at OFS: osf.io/cd35u.
